# Presence of Mobile Tigecycline Resistance Gene *tet*(X4) in Clinical Klebsiella pneumoniae

**DOI:** 10.1128/spectrum.01081-21

**Published:** 2022-02-09

**Authors:** Weishuai Zhai, Yingxin Tian, Mi Lu, Muchen Zhang, Huangwei Song, Yulin Fu, Tengfei Ma, Chengtao Sun, Li Bai, Yang Wang, Dejun Liu, Ying Zhang

**Affiliations:** a Beijing Key Laboratory of Detection Technology for Animal-Derived Food Safety, College of Veterinary Medicine, China Agricultural University, Beijing, People’s Republic of China; b Department of Laboratory Medicine, First Medical Centre, Chinese PLA General Hospital, Beijing, People’s Republic of China; c National Health Commission Key Laboratory of Food Safety Risk Assessment, Food Safety Research Unit (2019RU014) of the Chinese Academy of Medical Science, China National Center for Food Safety Risk Assessment, Beijing, People’s Republic of China; Peking University People's Hospital

**Keywords:** *tet*(X4), *Klebsiella pneumoniae*, tigecycline resistance, IncFII

## Abstract

The recently emerged plasmid-mediated tigecycline resistance gene *tet*(X4) has mainly been detected in Escherichia coli but never in Klebsiella pneumoniae. Herein, we identified a clinical K. pneumoniae isolate that harbored the *tet*(X4) gene located on a non-self-transferable IncFII-type plasmid, which could be cotransferred with a conjugative plasmid to E. coli C600. The extending of bacterial species carrying *tet*(X4) suggested the increasing risk of spreading mobile tigecycline resistance genes among important pathogens in clinical settings.

**IMPORTANCE** Tigecycline, the first member of glycylcycline class antibiotic, is often considered one of the effective antibiotics against multidrug-resistant (MDR) infections. However, the emergence and wide distribution of two novel plasmid-mediated tigecycline resistance genes, *tet*(X3) and *tet*(X4), pose a great threat to the clinical use of tigecycline. The newly *tet*(X) variants have been identified from multiple different bacterial species, but the *tet*(X) variant in the Klebsiella pneumoniae strain has been reported only once before. In this study, we identified a clinical K. pneumoniae isolate that harbored a non-self-transferable *tet*(X4)-carrying plasmid. This plasmid has never been found in other *tet*(X4)-harboring strains and could be cotransferred with a conjugative plasmid to the recipient strain. Our findings indicate that the *tet*(X4) gene breaks through its original bacterial species and spreads to some important nosocomial pathogens, which posed a serious threat to public health.

## OBSERVATION

The emergence of two novel *tet*(X) variants *tet*(X3) and *tet*(X4) constitute a serious threat to human health (1, 2). To date, *tet*(X3) and *tet*(X4) have been identified from over 10 different bacterial species (1, 3–5). The *tet*(X3) gene is predominantly identified in Acinetobacter species (1, 3), while the *tet*(X4) gene is dominantly found in Escherichia coli (1–3). However, the presence of *tet*(X) variants among Klebsiella pneumoniae isolates is rare, with sporadic cases (1). K. pneumoniae has the ability to carry acquired resistance to multiple antimicrobials, especially colistin (6) and carbapenems (7), which are often considered the last-line antimicrobial agents for treating multidrug-resistant infections. Here, to the best of our knowledge, we report for the first time the plasmid-mediated tigecycline resistance gene *tet*(X4) in a clinical K. pneumoniae strain.

During our 2019 surveillance study in a hospital located in Beijing, one isolate KP85 recovered from a fecal sample of a female inpatient was separated on CHROMagar orientation agar plates (CHROMagar, France) containing tigecycline (4 mg/L) and identified as K. pneumoniae by a matrix-assisted laser desorption ionization–time of flight (MALDI-TOF) mass spectrometry apparatus (Bruker, Germany) and positive for *tet*(X4) gene by PCR and Sanger sequencing (8). The susceptibility testing using broth microdilution method and interpreted by Clinical and Laboratory Standards Institute (9) and the European Committee on Antimicrobial Susceptibility Testing (10) criteria revealed that KP85 was resistant to sulfamethoxazole-trimethoprim, florfenicol, and all tested tetracyclines (tigecycline, oxytetracycline, tetracycline, chlortetracycline, doxycycline, and minocycline) but sensitive to gentamicin, cefotaxime, ciprofloxacin, meropenem, and colistin (Table S1). Meanwhile, it also exhibits resistance to the newly Food and Drug Administration (FDA)-approved tetracycline antibiotics eravacycline and omadacycline (minimal inhibitory concentration [MIC] = 32 mg/L). Conjugation assay was performed by the filter mating assay on LB agar with E. coli C600 (streptomycin resistant) as the recipient. The tigecycline resistance profile can be transferred from KP85 to E. coli C600 by conjugation (Table S1), with a transfer frequency of ∼10^−8^, suggesting the transconjugant named TCKP85-1, carrying a *tet*(X4)-harboring plasmid.

**FIG 1:**
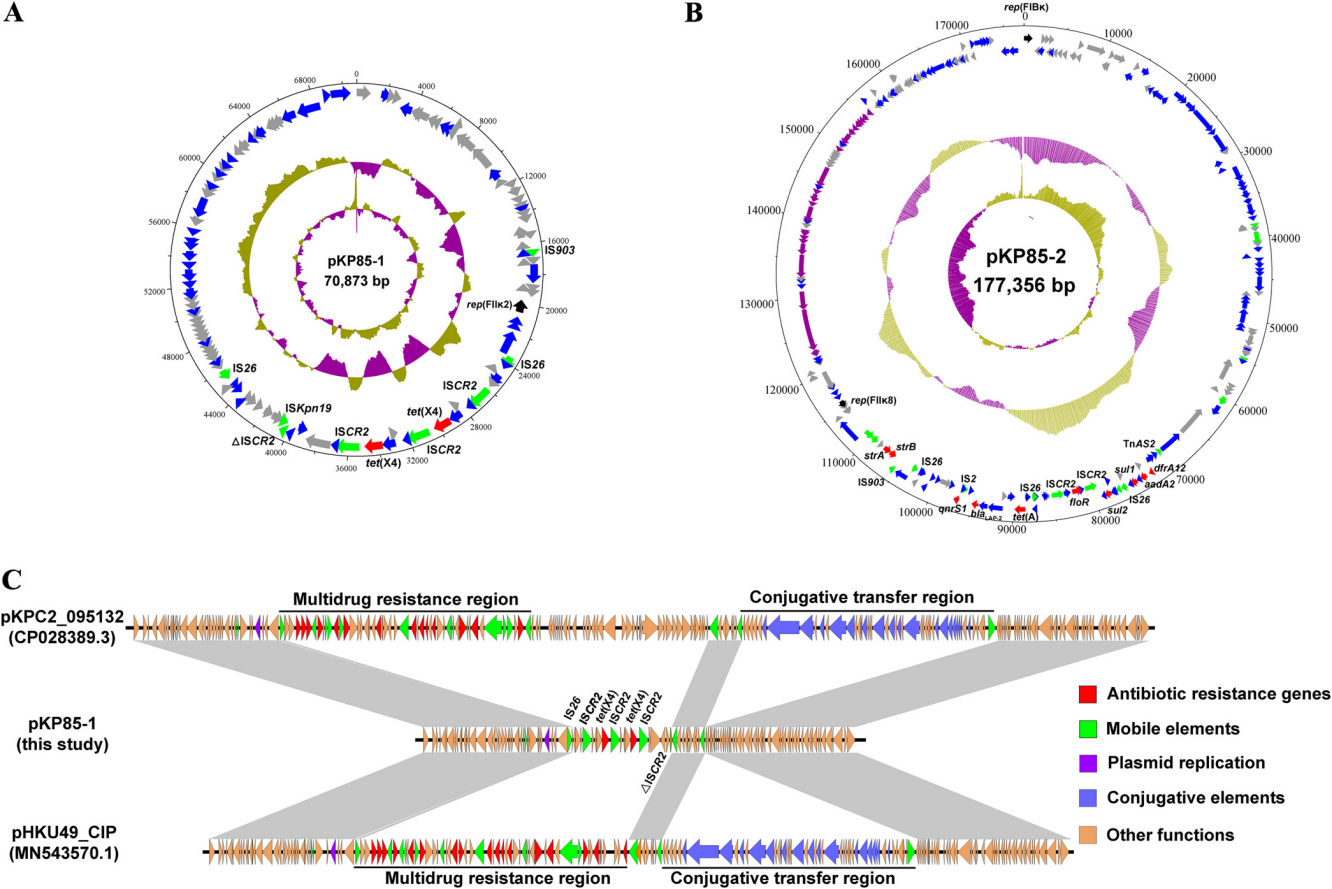
Genetic structure and comparative genomic analysis of plasmids recovered from K. pneumoniae strain KP85. (A, B) Genetic structure of plasmid pKP85-1 (A) and pKP85-2 (B) from K. pneumoniae KP85. Inner circle, GC skew; middle circle, G+C content. The arrows in the outer circle present the position and orientation of open reading frames (ORFs). Genes with different functions are labeled with different colors: black arrows represent replicon genes, green arrows represent mobile elements, red arrows represent antibiotic resistance genes, purple arrows represent conjugative elements, gray arrows represent hypothetical protein, and blue arrows represent other functional genes. (C) Comparative analysis of pKP85-1 with two online sequences. The light gray-shaded regions show more than 99% sequence identity. The arrows indicate gene orientations. Δ indicates a truncated gene, and different colors represent different categories of genes.

Genomic DNA of donor K. pneumoniae KP85 and transconjugant E. coli TCKP85-1 was extracted to characterize the genetic structure of the *tet*(X4)-harboring bacteria by using a TIANamp bacteria DNA kit (Tiangen, China). The genomes were sequenced using a Illumina Hiseq platform and Oxford Nanopore MinION. Each of the genomes was assembled using hybrid Illumina-Nanopore assemblies of Unicycler (version 0.4.8) and annotated using the RAST online annotation tool (https://rast.nmpdr.org/). Kleborate (version 2.1.0, https://github.com/katholt/Kleborate) was used to determine sequence type (ST), O:K locus profiles, and virulence genes. The antimicrobial resistance (AMR) determinants and plasmid replicon types were acquired in ResFinder and PlasmidFinder database at the Center for Genomic Epidemiology (CGE) website (https://cge.cbs.dtu.dk/services), respectively. We obtained a 5.39-MB complete genome of KP85, including a 5.19-MB circular chromosome, a 70,873-bp IncFII_K_-type plasmid pKP85-1 (Fig. 1A), and a 177,356-bp IncFIB_K_/FII_K_ hybrid plasmid pKP85-2 (Fig. 1B). The MLST result showed that K. pneumoniae KP85 belonged to a rare sequence type, ST534, which was identified in only one K. pneumoniae isolate recovered from a blood sample in Vietnam (11) (K. pneumoniae 131211-14450, accession no. ERR2586423) in 2011. KP85 had the same K locus (KL164) and O locus (OL102) as 131211-14450, while both strains were absent for known acquired virulence determinants and have a virulence score of 0 of 5, according to the Kleborate virulence score system. The difference among two strains is that KP85 possessed more AMR genes than 131211-14450. In our case, 13 resistance genes other than *tet*(X4) were identified in KP85, including genes resistance to aminoglycoside (*strA*, *strB*, and *aadA2*), β-lactam (*bla*_SHV-11_ and *bla*_LAP-2_), fluoroquinolone (*oqxAB* and *qnrS1*), tetracycline [*tet*(A)], fosfomycin (*fosA*), phenicol (*floR*), sulfonamide (*sul1* and *sul2*), and trimethoprim (*dfrA12*), 10 of which (except for *oqxAB*, *fosA*, and *bla*_SHV-11_) were located on the larger plasmid pKP85-2. The *tet*(X4) gene was the only resistance gene located on small plasmid pKP85-1, which is inconsistent with previous observations that *tet*(X4) is typically located on plasmids carrying multiple resistance genes (12–15), especially those carrying the *floR* and *tet*(A) genes.

Subsequently, a BLASTN search was conducted using the *tet*(X4)-harboring plasmid pKP85-1 as a reference sequence against the NCBI database. This sequence had 78% query coverage and more than 99.9% identity to several multidrug-resistant plasmids recovered from clinical K. pneumoniae strains, such as a 166,034-bp pKPC_0915132 (accession no. CP028389) and a 140,566-bp pHKU49_CIP (accession no. MN543570). These plasmids have almost identical backbone regions but distinct resistance-determining regions (Fig. 1C), indicating that the formation of pKP85-1 might be related to the recombination of variable regions in this type of plasmid. Two copies of *tet*(X4) were identified to be tandem repeated in the resistance-determining region of pKP85-1 as the form of IS*CR2-orf1-orf2*-*abh*-*tet*(X4)-IS*CR*2 (Fig. 1A). Previous studies have shown that the tandem repeated *tet*(X) genes are common among Enterobacteriaceae (13, 15), and this structure is generally considered to be related to the rolling-circle transposition of insertion sequence IS*CR*2 (16). Furthermore, the IS*26* and ΔIS*CR2* were found to be located upstream and downstream of the tandem repeated region, respectively (Fig. 1A and C), indicating that the transfer of *tet*(X4) in K. pneumoniae is highly likely to relate with the horizontal gene transfer of IS*CR2* and IS*26*. Although pKP85-1 lacks the conjugative elements and is considered a non-self-transferable plasmid, the plasmid pKP85-2 contains an intact cluster of type IV secretion system (17), and the transfer of the *tet*(X4)-carrying pKP85-1 might be with the assistance of pKP85-2. We further analyzed the plasmid recovered from transconjugant E. coli TCKP85-1 and identified that the *tet*(X4)-carrying region was pruned from the plasmid sequence because of tandem repeats (Fig. S1A). Thus, the whole genome reassembled by using the long-read data identified one chromosome and one ∼220-kb plasmid pTCKP85, and the plasmid carried a tandem repeated sequence with three copies of the *tet*(X4) gene (Fig. S1B and S2). The pTCKP85 shares high sequence identity (more than 99.88%) with pKP85-1 and pKP85-2. Further alignment of three plasmids revealed that pTCKP85 was formed by IncFII_K_ plasmid pKP85-1 and IncFIB_K_/FII_K_ hybrid plasmid pKP85-2 during conjugation, and its formation may be associated with the homologous recombination of IS*26* (Fig. S2). The strictly narrow-host-range IncFII_K_-type plasmids are major prevalent in K. pneumoniae (7), whereas the coexistence with other replicons (such as IncN) contributes to broadening the host range of IncFII_K_ plasmids (18, 19). The phylogenetic tree of IncFII_K_ alleles constructed by the maximum likelihood method by MEGA X indicated that the alleles in pKP85-1 and pKP85-2 belong to IncFII_K2_ and IncFII_K8_, respectively (Fig. S3), which might be the reason that even if the IncFII_K_-type replicon is identified in both plasmids, they can still form a recombinant plasmid pTCKP85. Additionally, the IncFII_K_ replicon, when coexisting with IncFIA and/or IncFIB replicons, does not participate in the initiation of plasmid replication (7, 18), which further explains why the two IncFII_K_ replicons can coexist in one plasmid. At present, the *tet*(X4)-harboring IncF family plasmids were observed commonly in the form of hybrid plasmid (12–15), which may be a major contributor to assist the *tet*(X4) gene in breaching the biological boundaries and spreading to different bacterial genus and species.

Moreover, to further determine whether plasmid reorganization occurred in all transconjugants, the whole-genome sequence of additional 19 transconjugants was then performed using an Illumina sequencing technique. The plasmid replicon analysis of 20 transconjugants (containing TCKP85-1) showed significant plasmid diversity (Table S2). A total of five different combinations of plasmid replicon were found in these transconjugants, including IncFIB_K_/FII_K2/_FII_K8_ (*n* = 11), IncFIB_K_/FII_K8_ (*n* = 4), IncFIB_K_/FII_K2_ (*n* = 2), IncFII_K2_/FII_K8_ (*n* = 2), and IncFIB_K_ (*n* = 1). In addition, five types of AMR gene profile were observed, which has no correlation with the plasmid replicon types (Table S2), indicating that a series of complex plasmid reorganizations occurred in the process of conjugation. To determine the gene deletions on plasmids after reorganization, genomes of all transconjugants were compared to pKP85-1 and pKP85-2 using BRIG (BLAST Ring Image Generator, http://brig.sourceforge.net/) software (Fig. S4 and S5). Nearly all transconjugants exhibited some degree of plasmid sequence deletion, the majority of which occurred in the multidrug resistant (MDR) region of the pKP85-2. Notably, a wide variety of mobile elements were found in the MDR region of pKP85-2, in particular IS*CR2* and IS*26* (Fig. 1B). Specifically, IS*CR2* was identified upstream and downstream of the *floR* gene, while IS*26* was identified in three sites of this region and named IS*26*-*strB* (adjacent *strB*), IS*26*-*tet*(A) [adjacent *tet*(A)], and IS*26*-*sul1* (adjacent *sul1*), respectively. These results indicated that the presence of these mobile elements might be the main reason for the variety of plasmids in transconjugants. However, due to complex plasmid reorganization and limitation of the sequencing length, our current findings could not characterize all possible plasmid structures in tested transconjugants and mark its homologous regions. Nevertheless, our current data indicated that the mobile elements (especially IS*26*) play an important role in the recombination of the plasmid between K. pneumoniae KP85 and its transconjugants (Fig. S6). As a matter of fact, the IS*26*-mediated *tet*(X4)-bearing plasmid reorganization during conjugation has already been found in *tet*(X4)-harboring E. coli (13, 15).

In conclusion, the presence of the *tet*(X)-carrying K. pneumoniae is predictive of the *tet*(X4) gene breakthrough from its original bacterial species and contributes to the spread of tigecycline resistance among important nosocomial pathogens, which poses an increasing challenge to public health. Further epidemiological surveillance should be performed in clinical settings to help medical practitioners proposing more effective measures against the infections by important clinical tigecycline-resistant pathogens.

### Nucleotide sequence accession numbers.

The sequence data of K. pneumoniae KP85 has been submitted to NCBI under BioProject accession number PRJNA747748. The sequence data of all transconjugants were deposited in the figshare database (https://doi.org/10.6084/m9.figshare.17065496) for reference.
