# Supplementary material for: Presence of Mobile Tigecycline Resistance Gene tet(X4) in Clinical Klebsiella pneumoniae
Source: Microbiol Spectr. 2022 Feb 9;10(1):e01081-21. doi: 10.1128/spectrum.01081-21 (PMC8826827; doi:10.1128/spectrum.01081-21)
Supplement: SUPPLEMENTAL FILE 1 — Supplemental material. Download SPECTRUM01081-21_Supp_1_seq4.pdf, PDF file, 1.6 MB [file spectrum01081-21_supp_1_seq4.pdf]

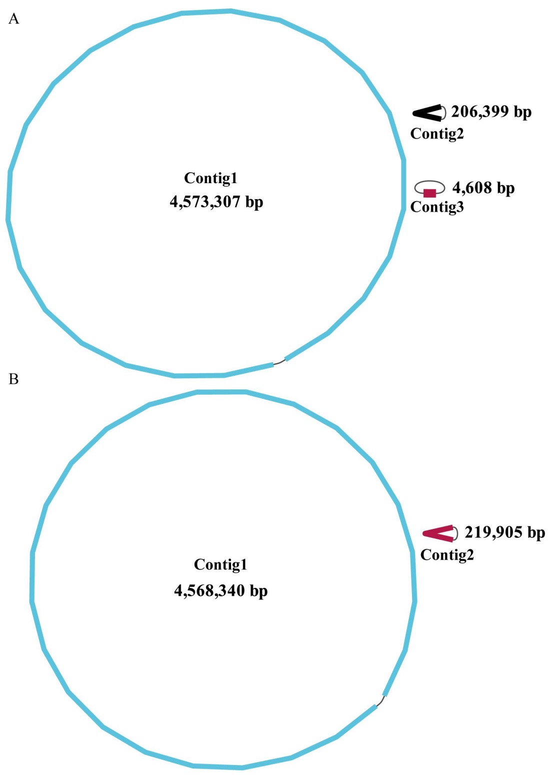

Figure S1 The simplified assembly graph of transconjugant *E. coli* TCKP85-1. (A) Hybrid assembly genome of *E. coli* TCKP85-1; (B) Long-read assembly genome of *E. coli* TCKP85-1. Blue bar represents the circular chromosome of *E. coli* TCKP85-1, red bar represents *tet*(X4)-harbouring circular contig. Assemblies were visualised using Bandage (https://github.com/rrwick/Bandage).

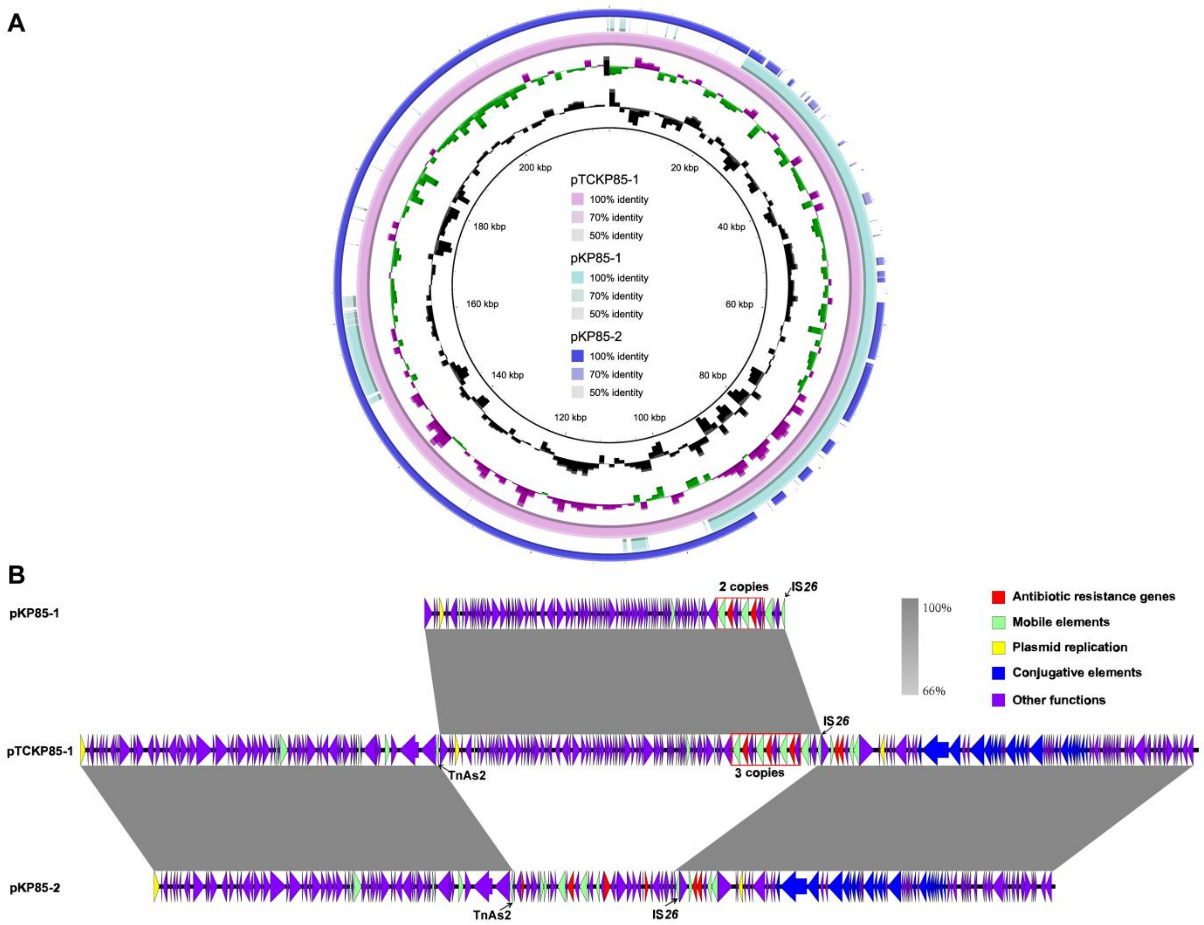

Figure S2 Comparative analysis of plasmids between donor and transconjugant strains. (A) Alignment of three plasmids using the BLAST Ring Image Generator (BRIG). The plasmids in KP85 and TCKP85-1 are indicated in light blue (pKP85-1), dark blue (pKP85-2) and pink (pTCKP85-1), respectively. (B) Alignment of three plasmids using EasyFig software. The arrows in different colours represent the function and direction of different genes, gray shading represents the sequence identity.

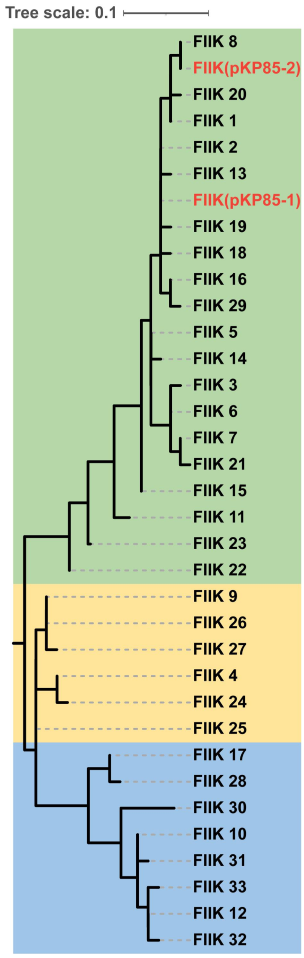

Figure S3 Phylogenetic tree of IncFII$_K$ alleles. Different colours indicate different IncFII$_K$ allele groups. Red numbering refers to the IncFII$_K$ alleles in this study. All IncFII$_K$ alleles used in the phylogenetic analysis were obtained from pMLST (http://pubmlst.org/plasmid/).

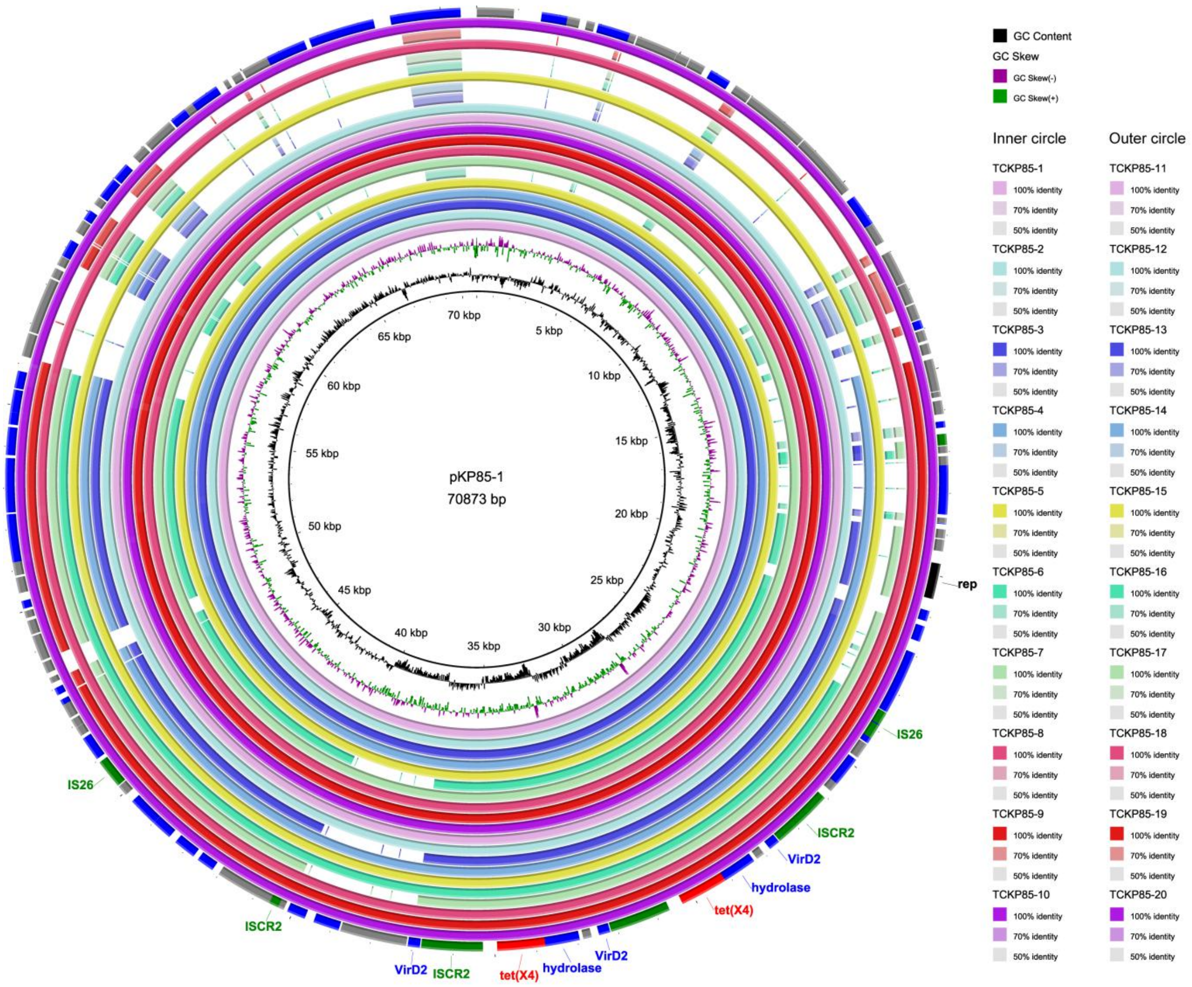

Figure S4 Comparative genome analysis of pKP85-1 with 20 transconjugant genomes. Genes with different functions are labeled with different colours on the outermost circle, black represents replicon genes, green represents mobile elements, reds represent antibiotic resistance genes, gray represents hypothetical protein and blues represent other functional genes.

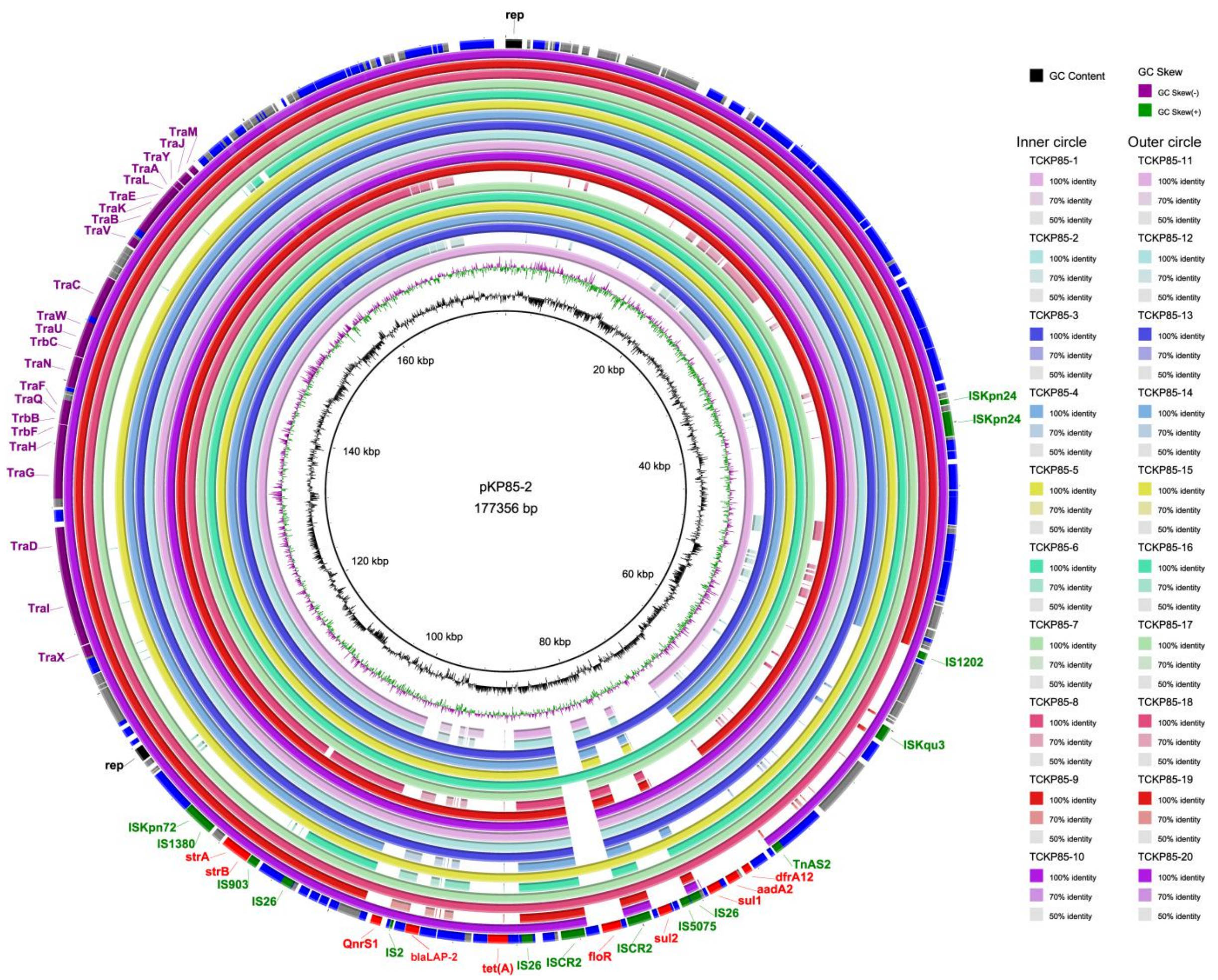

Figure S5 Comparative genome analysis of pKP85-2 with 30 transconjugant genomes. Genes with different functions are labeled with different colours on the outermost circle, black represent replicon genes, green represent mobile elements, red represent antibiotic resistance genes, purple represent conjugative elements, gray represent hypothetical protein and blue represent other functional genes.

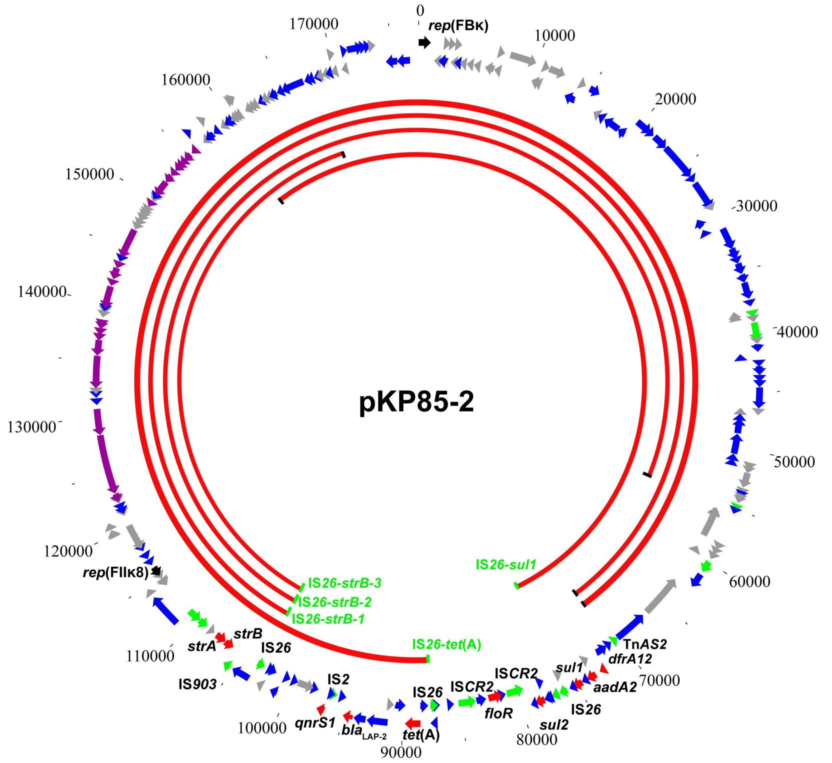

Figure S6 A schematic graph of several possible recombination mechanisms (IS*26*-mediated) in pKP85-2. The red ring represents the sequence retained after plasmid recombination. Genes with different functions are labeled with different colours, black arrows represent replicon genes, green arrows represent mobile elements, red arrows represent antibiotic resistance genes, purple arrows represent conjugative elements, gray arrows represent hypothetical protein and blue arrows represent other functional genes. This figure is deduced based on Figure S5 and Table S2.

Table S1 Susceptibility of *K. pneumoniae* KP85 and its transconjugant to commonly used antibiotics.

**Strains**	**Descriptions**	**TGC**	**ERA**	**OMA**	**OTC**	**TET**	**CTE**	**DOX**	**MIN**	**FFC**	**SXT**	**GEN**	**CTX**	**CIP**	**MEM**	**COL**
K. pneumoniae KP85	Donor	32	32	32	>128	>128	128	64	64	>32	>32	0.5	0.06	0.5	<0.5	<0.5
E. ***coli*** C600	Recipient	0.25	0.25	1	2	2	2	1	0.5	2	<1	0.5	0.03	0.03	<0.5	<0.5
E. coli TCKP85	Transconjugant	16	16	32	>128	128	128	64	64	2	<1	0.5	0.03	0.5	<0.5	<0.5

TGC, Tigecycline; ERA, Eravacycline; OMA, Omadacycline; OTC, Oxytetracycline; TET, Tetracycline; CTE, Chlortetracycline; DOX, Doxycycline; MIN, Minocycline; FFC, Florfenicol; SXT, trimethoprim/sulfamethoxazole; GEN, Gentamicin; CTX, Cefotaxime; CIP, Ciprofloxacin; MEM, Meropenem; COL, Colistin.

Table S2 Plasmid replicon types and AMR gene profiles of 20 transconjugants.

Number	Resistance genes	Plasmid replicon
TCKP85-2	*strA*, *strB*, *tet*(X4)	IncFII$_{K2}$, IncFII$_{K8}$
TCKP85-8	*strA*, *strB*, *tet*(X4)	IncFII$_{K2}$, IncFII$_{K8}$
TCKP85-19	*strA*, *strB*, *tet*(X4)	IncFIB$_{K}$, IncFII$_{K2}$
TCKP85-14	*strA*, *strB*, *tet*(X4)	IncFIB$_{K}$, IncFII$_{K2}$, IncFII$_{K8}$
TCKP85-1	*strA*, *strB*, *tet*(X4)	IncFIB$_{K}$, IncFII$_{K2}$, IncFII$_{K8}$
TCKP85-16	*aadA2*, *dfrA12*, *sul1*, *tet*(X4)	IncFIB$_{K}$
TCKP85-4	*strA*, *strB*, *qnrS1*, *tet*(A), *tet*(X4), *bla*$_{LAP-2}$	IncFIB$_{K}$, IncFII$_{K2}$, IncFII$_{K8}$
TCKP85-5	*strA*, *strB*, *qnrS1*, *tet*(A), *tet*(X4), *bla*$_{LAP-2}$	IncFIB$_{K}$, IncFII$_{K2}$, IncFII$_{K8}$
TCKP85-9	*strA*, *strB*, *qnrS1*, *tet*(A), *tet*(X4), *bla*$_{LAP-2}$	IncFIB$_{K}$, IncFII$_{K2}$, IncFII$_{K8}$
TCKP85-20	*strA*, *strB*, *qnrS1*, *tet*(A), *tet*(X4), *bla*$_{LAP-2}$	IncFIB$_{K}$, IncFII$_{K2}$, IncFII$_{K8}$
TCKP85-10	*aadA2*, *strA*, *strB*, *dfrA12*, *qnrS1*, *sul1*, *sul2*, *tet*(A), *tet*(X4), *bla*$_{LAP-2}$	IncFIB$_{K}$, IncFII$_{K2}$, IncFII$_{K8}$
TCKP85-11	*aadA2*, *strA*, *strB*, *dfrA12*, *qnrS1*, *sul1*, *sul2*, *tet*(A), *tet*(X4), *bla*$_{LAP-2}$	IncFIB$_{K}$, IncFII$_{K2}$, IncFII$_{K8}$
TCKP85-12	*aadA2*, *strA*, *strB*, *dfrA12*, *qnrS1*, *sul1*, *sul2*, *tet*(A), *tet*(X4), *bla*$_{LAP-2}$	IncFIB$_{K}$, IncFII$_{K2}$, IncFII$_{K8}$
TCKP85-3	*aadA2*, *strA*, *strB*, *dfrA12*, *qnrS1*, *sul1*, *sul2*, *tet*(A), *tet*(X4), *bla*$_{LAP-2}$	IncFIB$_{K}$, IncFII$_{K2}$
TCKP85-7	*aadA2*, *strA*, *strB*, *dfrA12*, *qnrS1*, *sul1*, *sul2*, *tet*(A), *tet*(X4), *bla*$_{LAP-2}$	IncFIB$_{K}$, IncFII$_{K2}$, IncFII$_{K8}$
TCKP85-13	*aadA2*, *strA*, *strB*, *dfrA12*, *qnrS1*, *sul1*, *sul2*, *tet*(A), *tet*(X4), *bla*$_{LAP-2}$	IncFIB$_{K}$, IncFII$_{K8}$
TCKP85-6	*aadA2*, *strA*, *strB*, *dfrA12*, *qnrS1*, *sul1*, *sul2*, *tet*(A), *tet*(X4), *bla*$_{LAP-2}$, *floR*	IncFIB$_{K}$, IncFII$_{K8}$
TCKP85-17	*aadA2*, *strA*, *strB*, *dfrA12*, *qnrS1*, *sul1*, *sul2*, *tet*(A), *tet*(X4), *bla*$_{LAP-2}$, *floR*	IncFIB$_{K}$, IncFII$_{K8}$
TCKP85-18	*aadA2*, *strA*, *strB*, *dfrA12*, *qnrS1*, *sul1*, *sul2*, *tet*(A), *tet*(X4), *bla*$_{LAP-2}$, *floR*	IncFIB$_{K}$, IncFII$_{K8}$
TCKP85-15	*aadA2*, *strA*, *strB*, *dfrA12*, *qnrS1*, *sul1*, *sul2*, *tet*(A), *tet*(X4), *bla*$_{LAP-2}$, *floR*	IncFIB$_{K}$, IncFII$_{K2}$, IncFII$_{K8}$
